# A novel projection data domain material decomposition method for dual-energy CT and its impact on the accuracy of attenuation values

**DOI:** 10.1088/1361-6560/ae4163

**Published:** 2026-02-16

**Authors:** Viktor Haase, Frédéric Noo, Karl Stierstorfer, Andreas Maier, Michael McNitt-Gray

**Affiliations:** 1 Siemens Healthineers AG, Forchheim, Germany; 2 Department of Computer Science, Friedrich-Alexander-Universität Erlangen-Nürnberg, Erlangen, Germany; 3 Department of Radiology and Imaging Sciences, University of Utah, Salt Lake City, UT, United States of America; 4 Department of Radiology, David Geffen School of Medicine, University of California, Los Angeles, CA, United States of America

**Keywords:** dual-energy computed tomography (DECT), data-based material decomposition, analytical energy response model, Hounsfield unit (HU) accuracy, quantitative CT imaging

## Abstract

*Objective.* Despite major advances in dual-energy computed tomography (CT), obtaining accurate attenuation values for quantitative applications remains a technical challenge. To address this topic, we introduce a novel projection data domain material decomposition method that is an extension of an approach we recently proposed for beam hardening correction in single energy CT. *Approach.* The proposed method employs object-specific scatter correction and an analytical energy response model. We compare its performance to image-based material decomposition on accuracy of attenuation values using the American College of Radiology (ACR) CT accreditation phantom, scanned with consecutive low and high energy axial scans in centered and off-centered positions. Accuracy is assessed across the five inserts, and the images are analyzed for beam hardening artifacts and noise. Additionally, we assess the usefulness of object-specific scatter correction, and we assess performance over conventional data domain material decomposition and for anthropomorphic abdomen phantom imaging. *Main results.* In the ACR phantom, the proposed method yielded a significant improvement in accuracy of the attenuation values, particularly at low energies ($ \lt\!\!70$ keV), and an important reduction in beam hardening artifacts. While similarly high accuracy was achieved for water, quantitative error within the non-water inserts was lower and more uniform across the 30–140 keV range, especially in the more challenging off-centered positioning of the phantom. Noise showed expected parabolic behavior, but with minimum at lower keV, which may be clinically advantageous. Object-specific scatter correction was shown to prevent major artifacts. Advantages over conventional data-domain decomposition clearly appeared when only a standard phantom is available to calibrate the latter. Lastly, the proposed method was shown to perform well, without any changes, in the more complex scenario of abdominal phantom imaging. *Significance.* This work demonstrates that data-based material decomposition using an analytical energy response model with object-specific scatter correction offers a promising pathway to improve accuracy of CT attenuation values.

## 1. Introduction

X-ray computed tomography (CT) is one of the most important imaging modality in radiology. Clinically, CT is used both for disease detection and as a quantitative tool for disease classification and management. Most often, quantitative analysis is, however, focused on size measurements only, disregarding the attenuation values. To enable reliable clinical utilization of CT attenuation values over any region of the human body, it is well appreciated that a dual-energy (DE) data acquisition solution is needed. This is because all human tissues can be well-represented by linearly combining the x-ray attenuation coefficients of two materials, and finding the two coefficients of this combination requires two data sets that differ in terms of energy spectrum (Alvarez and Macovski 1976). Many ways of performing DE data acquisition have been implemented on modern clinical scanners, including sequential spiral scans (Schmidt and Flohr 2022), fast kVp switching (Xu *et al* 2009, Chandra and Langan 2010), dual source CT (Flohr *et al* 2006, Johnson *et al* 2007), dual layer detectors (Carmi *et al* 2005, Ehn *et al* 2018), and photon-counting detectors (Flohr *et al* 2020). Reviews of these methods can be found in McCollough *et al* (2015), Goo and Goo (2017), and Richter *et al* (2022).

A vast body of literature has demonstrated clinical value of DE computed tomography (DECT) over most anatomical regions of the human body, for both disease detection and classification. The idea that DECT would enable robust clinical utilization of attenuation values has, however, not yet been realized (Treb *et al* 2025), except perhaps for some specific problems like kidney stone differentiation (Israel and Bosniak 2005, Davenport *et al* 2011, Krishna *et al* 2017) and visceral fat measurements (Duman *et al* 2006, Shuster *et al* 2012), which supports strong clinical potential for the CT attenuation values.

It turns out that obtaining accurate CT attenuation values using DE data acquisition is not straightforward, as all solutions come with major physics-based challenges such as, for example, cross-scatter for dual source systems (Erath *et al* 2021), variable spectra for fast kVp-switching (Xu *et al* 2009), and high noise in one of the spectral channel for dual-layer detectors and photon-counting detectors (McCollough *et al* 2020). In 2011, shortly after DECT had become widely commercially available, Goodsitt *et al* (2011) investigated the accuracy of CT attenuation values in mono-energetic images created with DE data acquisition. While encouraging results were shown, the authors emphasized that important limitations remained. This has been re-iterated by others (Yu *et al* 2012) and remains an open problem (Treb *et al* 2025), although there is evidence of progress being possibly made (Sellerer *et al* 2018).

As discussed in Rit *et al* (2020), there are three main ways to perform image reconstruction from DE data: one-step image reconstruction, projection data domain material decomposition, and image domain material decomposition. See, e.g. Maaß *et al* (2011), Malusek *et al* (2017), Xue *et al* (2019), Geng *et al* (2020), Ren *et al* (2021), and Chen *et al* (2025) for some recent interesting technical advances regarding these. The one-step image reconstruction is conceptually appealing and promising, but unfortunately remains too computationally demanding for fast clinical workflow. Implementation of one-step image reconstruction can be done in a mathematically rigorous manner through formulation of an objective function to be minimized (as, e.g. in Barber *et al* (2016)), or in a more empirical manner using iterations that involve repeated filtered-back projection reconstructions (as, e.g. in Maaß *et al* (2011), Malusek *et al* (2017)). Note that the rigorous manner leads to a non-convex optimization problem that can be affected by robustness issues due to local minima. Note also that the empirical manner allows using much fewer iterations, which may position it closer to future clinical translation. The projection data domain material decomposition, called data domain for short in the rest of the manuscript, was originally proposed in Alvarez and Macovski (1976). It is geometrically more constraining than the other two ways, as it assumes spatial alignment of the DE measurements, which is not compatible with some DE implementations like spiral data acquisition with dual source, and which requires interpolation that affects accuracy for other implementations like dual-layer detectors and fast-kVp switching. It also involves solving non-linear equations that can be numerically challenging (Manhart *et al* 2014, Rit *et al* 2020). Some works have offer stable solutions by solving these equations as an optimization problem with regularization constraints (e.g. Ying *et al* (2006), Brendel *et al* (2016)); however, it is important to realize that such constraints can introduce bias in the image reconstruction (Rit *et al* 2020). Altogether, the constraints that affect one-step image reconstruction and data domain material decomposition have led at least one vendor to prefer the image domain approach. However, image domain material decomposition is suboptimal for quantitative analysis based on CT attenuation values, because images created from such a decomposition suffer from beam hardening errors in the same way as single energy CT does (McCollough *et al* 2020).

To the best of our knowledge, the preferred approach for data domain material decomposition is to scan different lengths of two materials to obtain calibration data and to use this data to create a mathematical relationship that enables converting each pair of DE measurements into two lengths, one for each material. These material lengths can be reconstructed to obtain material maps or recombined to create mono-energetic data from which mono-energetic images are reconstructed. This approach is a direct extension of the commonly-used water-based beam hardening correction. As such, it suffers as well from the issue that scatter cannot be disentangled from beam hardening; hence the method corrects in some ways for both beam hardening and scatter. Also, the calibration data does not account for negative material lengths, which can result, for example, in using fitted polynomials with extrapolation (e.g. to represent fat from water and bone).

Recently, we revisited the problem of single material (including water) correction for beam hardening, which led us to develop a method that disentangles scatter from beam hardening, using a simple object-specific scatter correction together with an analytical energy response model for beam hardening correction (Haase *et al* 2022). Evaluation of this method showed noticeable improvements in terms of CT number accuracy and reproducibility (Haase *et al* 2022). However, and not unexpectedly, these improvements were modest near bone structures like the spine. In this work, we explore extension of this approach to data domain DE material decomposition and compare this extension with state-of-the-art image domain material decomposition, towards the long term goal of achieving accurate CT attenuation values. An early version of this extension was presented along with some preliminary evaluations at two conferences (Haase *et al* 2021, 2022). Conceptually, our approach follows the footsteps of Ying *et al* (2006) where adaptive scatter subtraction was shown to be valuable for non-destructive testing using DECT, although our approach uses different algorithmic steps and focuses on clinical CT scanners. Our work also follows the suggestion in Goodsitt *et al* (2011) that scatter correction should continue to be investigated to improve quantitative accuracy of CT attenuation values.

The paper is organized as follows. Section 2 details the theoretical foundations and implementation of both image-based and the proposed data-based material decomposition methods. Sections 3 and 4 outline the experimental setups used for evaluation, including scanning parameters, phantom description, and figures of merit. Sections 5 and 6 present and compare the various results we obtained. Section 7 discusses the implications of the findings, addresses limitations, and explores the potential clinical relevance. Finally, section 8 concludes the paper and outlines directions for future research.

## 2. Theory

The most common methods to create mono-energetic images from DECT data are image-based and data-based material decomposition approaches. In this work, we are comparing both approaches using a specific realization of each of them. These realizations are described in this section. All image reconstruction steps mentioned herein correspond to applying the classical filtered backprojection formula with parallel-beam rebinning.

### 2.1. Image-based material decomposition

Here, the material decomposition takes place in the image domain, by processing the image reconstructed from the high-energy (HE) projection dataset together with the image reconstructed from the low-energy (LE) projection dataset. The image processing steps that we used for the decomposition were adapted from Ren *et al* (2021), and are representative of implementation steps employed by vendors. The key underlying principle of image-based decomposition is to find an effective energy at which the low energy image can be approximately interpreted as a mono-energetic image, and the same for the high energy image.

**Figure 1.**
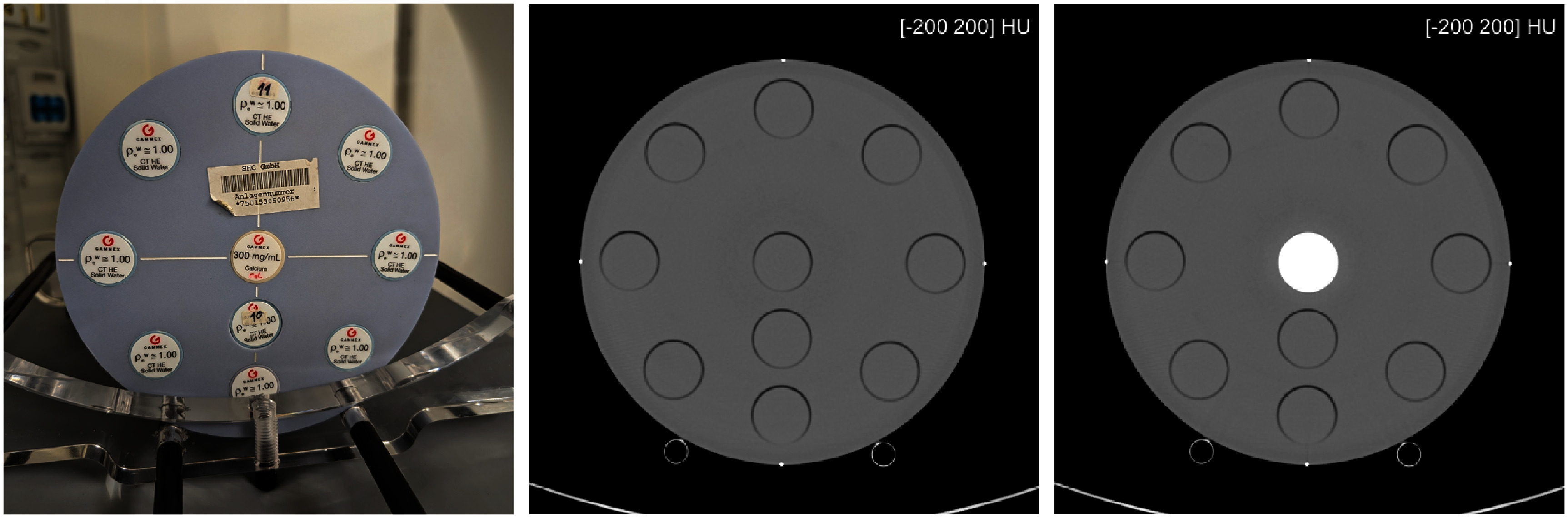
Calibration phantom for image-based material decomposition. (Left) Photo of the head portion of the Multi-Energy CT Phantom of Sun Nuclear (Melbourne, FL, USA), which was used in our study. The phantom is here placed on the patient bed within the gantry opening of the scanner. (Middle) Reconstructed image of a 120 kV scan showing the calibration phantom with all inserts made of water-equivalent material. (Right) Reconstructed image of a 120 kV scan showing the calibration phantom with a calcium insert in the central hole (concentration of calcium: 300 mg ml^−1^), while all other inserts are of water-equivalent material.

There were two steps. The first one was to scan a water-equivalent phantom with cylindrical inserts made of the two basis materials chosen for the material decomposition. We refer to this phantom as the calibration phantom. We used the head portion of the Multi-Energy CT Phantom of Sun Nuclear (Melbourne, FL, USA), which consists of a cylinder of water-equivalent material (diameter: 20 cm) with holes enabling the insertion of up to ten cylindrical inserts (diameter: 2.86 cm). We created two configurations: one with all holes filled with water-equivalent inserts, and a second one with the central hole filled with a calcium insert (concentration: 300 mg ml^−1^) while all other holes were filled with water-equivalent inserts. We viewed the water-equivalent insert as one basis material and the calcium insert as the other basis material. See figure 1.

Once the LE and HE images were available, the mean attenuation value through the central insert was computed for both configurations of the phantom. The ratio of the mean values obtained for the water-equivalent and calcium inserts in the LE images was then compared with an energy-dependent ground truth (GT) ratio to identify an effective energy for the LE scans, called *ε*_LE_; and the same was done to obtain an effective energy for the HE scans, called *ε*_HE_. The GT ratio was obtained by using the known chemical composition of the water-equivalent and calcium inserts with the NIST tables of linear attenuation coefficients (NIST 2024). Two configurations of the phantom (one with water at the isocenter, and one with calcium at the isocenter) were preferred over a single configuration to ensure that beam hardening errors would not impact the mean attenuation value obtained for the water-equivalent insert.

The second step started with creating a $2 \times 2$ decomposition matrix:

\begin{equation*} D = \left[ \begin{array}{cc} \mu_\mathrm{water}\left(\varepsilon_{\mathrm{LE}}\right) &amp; \mu_\mathrm{calcium}\left(\varepsilon_{\mathrm{LE}}\right) \\ \mu_\mathrm{water}\left(\varepsilon_{\mathrm{HE}}\right) &amp; \mu_\mathrm{calcium}\left(\varepsilon_{\mathrm{HE}}\right) \end{array} \right]\end{equation*}

where $\mu_\mathrm{water}(\varepsilon)$ and $\mu_\mathrm{calcium}(\varepsilon)$ are the linear attenuation coefficients of the two basis materials at energy *ε*. At each pixel location, two attenuation values are available for the object scanned, one from the LE scan, and the other one from the HE scan. These two values were stacked to form a vector to which the inverse of *D* was applied. The result was a vector with two components, each of which represents the relative density of one of the basis materials. Linearly combining these two components with the linear attenuation coefficient of the two basis materials at any fixed energy created the pixel values of a mono-energetic image. Note that this is not the only way to implement this second step; for example, in Malusek *et al* (2017), the two-material decomposition is formulated using a $3 \times 3$ matrix inversion that allows interpreting the two unknowns as two normalized fractions-by-weight and mass density.

### 2.2. Data-based material decomposition

Here, the material decomposition takes place in the data domain, directly from the projection data of the LE and HE scans, before any image reconstruction step is applied. The projection data of the LE and HE scans are spatially-aligned, and the outcome of the decomposition is the line integrals of two functions that represent the relative densities along the basis materials. Image reconstruction is taking place after the decomposition and yields the relative densities over the pixels. As in the image-based approach, the mono-energetic image is obtained by linearly combining the relative densities with the linear attenuation coefficient of the two basis materials at the desired energy.

Our approach for the data domain decomposition was an extension from Haase *et al* (2022) where we proposed an improved data-based approach for single-material beam hardening correction that uses object-specific scatter subtraction together with an analytical energy response model for the source-detector spectrum. We started with LE and HE data that have not undergone any correction for beam hardening or scatter, and for which the following classical noise-free mathematical interpretation thus holds:

\begin{equation*} g_{m,i} = -\log \left\{\int_0^{E_{\mathrm{max}}} W_{m,i}\left(E\right) \, \exp\left(-\int_{-\infty} ^\infty \mu\left(E,{\underline x}_i\left(t\right)\right) \, \mathrm{d}t\right) \,\mathrm{d}E+ s_{m,i} \right\}\end{equation*}

where *g_m_* refers to the LE or HE data depending on the value (LE or HE) given to label *m*; *i* is an index that identifies the lines along which the measurements are taken; *t* is a Cartesian coordinate along the line of index *i*; $\mu(E,{\underline x}_i(t))$ is the linear attenuation coefficient of x-rays at position ${\underline x}_i(t)$ on the line of index *i*; $W_{m,i}$ is the normalized analytical energy response model; and $s_{m,i}$ is the scatter signal. The dependence of *W* on *i* is induced by the beam-shaping (bowtie) filter and other effects like the flying focal spot position.

**Figure 2.**
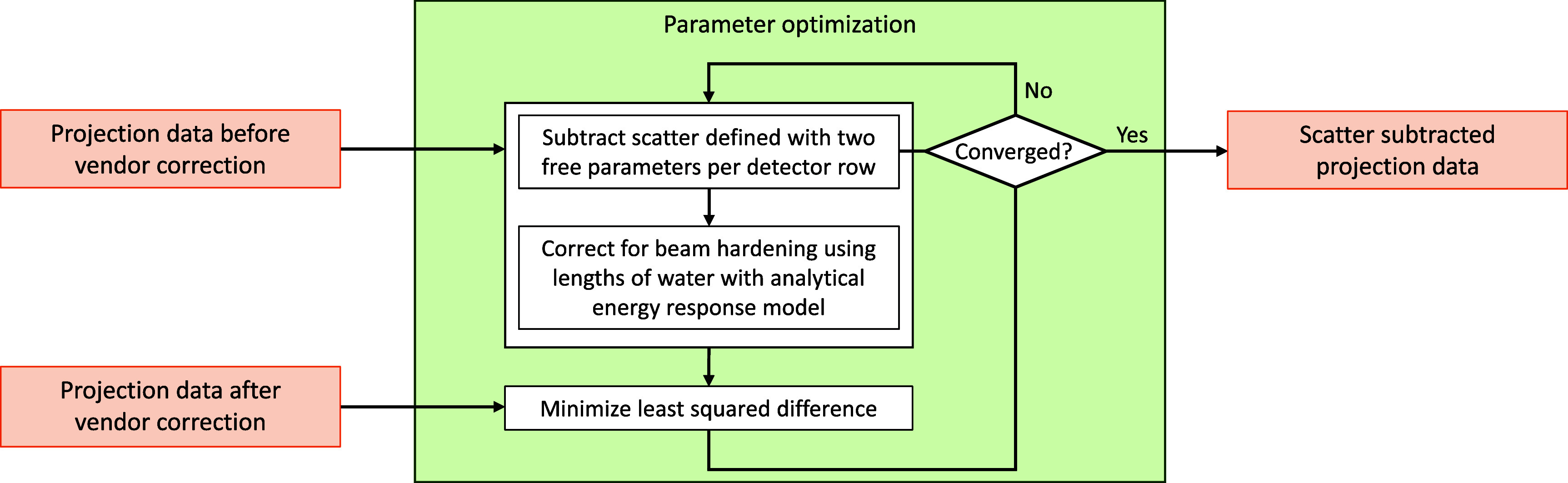
Scatter subtraction scheme. Our approach for data-based material decomposition requires first subtracting scatter signal from the projection data. This algorithm explains the approach chosen. Essentially, scatter is modeled with two free parameters per detector row and these parameters are found to match data that is jointly corrected for scatter and single material beam hardening by the vendor using a water-like calibration phantom. Detailed equations for the scatter model and the objective function can be found in Haase *et al* (2022). Adapted with permission from Haase *et al* (2022). © 2022 The Authors. Medical Physics published by Wiley Periodicals LLC on behalf of American Association of Physicists in Medicine. CC BY-NC-ND 4.0.

Next, we proceeded with a data pre-processing step that amounts to removing an estimate of the object-specific scatter (namely, $s_{m,i}$) from the LE scan, as well as from the HE scan, one after the other. This step was carried out using the exact same approach as in Haase *et al* (2022). Briefly, a simple object-specific model of scatter that only requires two free parameters per detector row is introduced. The free parameters are then obtained through an optimization process that aims to match, in average, two sets of projection data: (1) the set that the vendor uses for image reconstruction, which is empirically corrected for beam-hardening and scatter so as to ensure accurate reconstruction of a water-equivalent calibration phantom, (2) the measured data before the vendor correction to which object-specific scatter removal and then single material (namely, water) beam-hardening correction are individually applied to all lines (with scatter removal based on the simple object-specific model and beam-hardening correction based on knowledge of the analytical energy response $W_{m,i}$). See figure 2 for an illustration. The mathematical description of this step can be found in Haase *et al* (2022). The effectiveness of the optimization scheme used to estimate scatter is supported by the results in this same publication.

**Figure 3.**
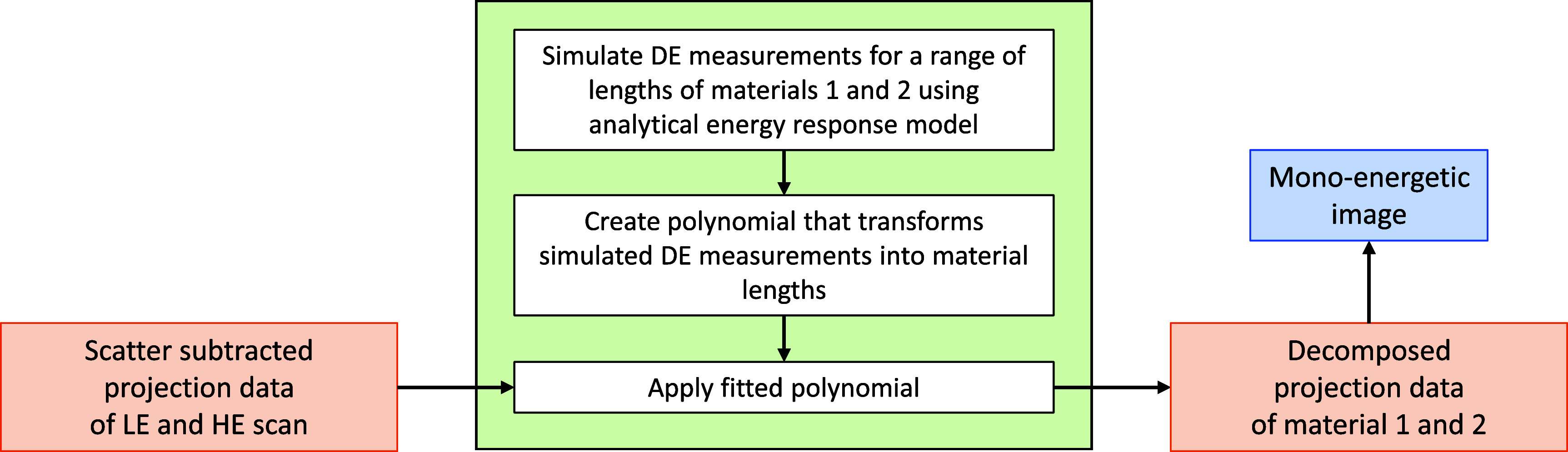
Data-based material decomposition approach. The projection data for the high and low energy scans are first individually corrected for scatter using the scheme of figure 2, then passed through two bivariate polynomials to obtain material lengths from which material images and mono-energetic images are obtained using classical filtered backprojection. The polynomial’s coefficients are found using a fitting procedure based on given material lengths and a known energy response model for the high and low energy scans; see Haase *et al* (2023) for details.

Once scatter has been removed, the data was interpreted as in equation (2) but without the $s_{m,i}$ term, and data-domain material decomposition was thus performed using an analytical energy response model for the LE and HE scans. Rather than numerically solving the two equations for each index *i*, we tabulated the solution using polynomial fittings as suggested in Lehmann *et al* (1981). That is, the desired line integrals were obtained by passing the LE and HE projection data through two polynomials. The coefficients of these polynomials were found through a least-square fitting procedure based on simulated measurements of various combinations of material lengths. See figure 3 for an illustration and see Haase *et al* (2023) for investigative details regarding the fitting procedure.

A more conventional approach for data domain material decomposition would be to use polynomials that are found from real measurements of known material lengths. This approach does not involve scatter subtraction, but naturally embeds some amount of correction for scatter within the polynomial fitting. Our approach was preferred for two reasons: (1) the scatter subtraction is object-specific, which was shown to be valuable in Haase *et al* (2022); (2) negative material lengths can be included within the polynomial fitting process, which is practical (e.g. the decomposition of fat on water and bone requires negative lengths) and maybe more robust than relying on extrapolation (which is the only way to address negative lengths when the fitting is based on real measurements of known material lengths).

## 3. Experiments on accuracy of attenuation values

This section provides all experimental details that were used to compare the performance of image-based material decomposition versus data-based material decomposition in terms of accuracy (bias) in reconstructed attenuation values.

### 3.1. Scan geometry and image reconstruction

All scans were performed at fixed bed position using a state-of-the-art CT scanner, namely the SOMATOM Definition Flash (Siemens Healthineers AG, Germany). The beam collimation of the axial scans was $32\,\times\,1.2$ mm. Full-scan reconstructions were performed with a slice thickness of 1.5 mm and a moderately smooth convolution kernel designed for quantitative applications that is called ‘D40s’. Quantitative kernels differ from non-quantitative kernels in that no edge enhancement is performed. The field-of-view was a circle of diameter equal to 250 mm, leading to a pixel size of 0.49 mm. Only the central slice of the reconstructed volumes was used in the analyses. DE imaging was achieved using consecutive scans collected at different tube voltages. Detailed information about all scan parameters including our settings for DE can be found in table 1.

**Table 1.** Parameters of scanner geometry.

Scan trajectory	360^∘^, fixed bed position
Number of projections	2304
Number of detector channels	736
Detector collimation	$32\,\times\,1.2$
Flying focal spot mode	in-plane
Source to detector distance	108.56 cm
Source trajectory radius	59.5 cm
Anode angle	7^∘^
Angular detector width	0.067864^∘^
LE tube settings	80 kV, 500 mAs
HE tube settings	120 kV, 300 mAs

To mitigate the effect of quantum noise, and thereby focus on accuracy, all scans were acquired 10 times. The 10 projection datasets were averaged together before performing image reconstruction, which was possible using an offline image reconstruction engine made available for research by Siemens Healthineers.

### 3.2. Phantom description

**Figure 4.**
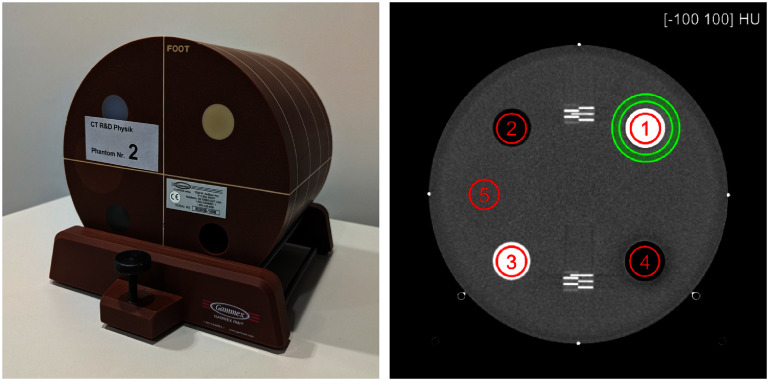
ACR phantom. (Left) Picture of the phantom. Module A, which was used for all experiments, can be seen at the forefront of the phantom. (Right) Example of reconstructed image though module A. The red circles show the five ROIs used for quantitative analysis within the inserts. The ROI labels are marking the different material inserts in ascending order from 1 to 5: bone, polyethylene, acrylic, air, and water. The two green circles delimitate a ring-shaped ROI (shaded in light green) used for quantitative assessment of beam hardening errors around the bone insert.

All experiments of this section 3 were conducted with module A of the ACR CT accreditation phantom (model 464, Gammex-RMI, USA). Module A is a cylinder of water-equivalent material with a diameter of 200 mm and a length of 40 mm. It contains five cylindrical inserts representing the x-ray attenuation behavior of air, bone, polyethylene, acrylic, and water; all inserts have a diameter of 25 mm, except the water cylinder, the diameter of which is 50 mm. In addition, the module shows horizontal ramps of wires spaced apart every 0.5 mm for slice thickness evaluation and four 1 mm diameter steel beads used to verify centering of the module in the direction of the patient bed. See figure 4. The chemical composition characteristics of each insert were provided by the phantom’s manufacturer.

### 3.3. Phantom positioning

**Figure 5.**
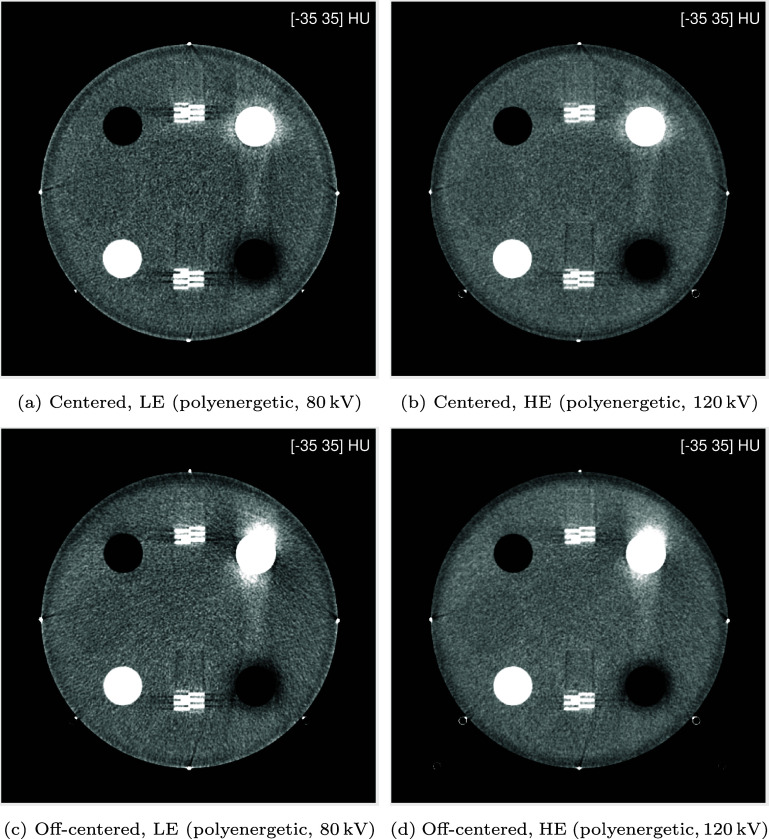
Images reconstructed from the LE and HE scans for the ACR phantom. (Top) centered positioning; (bottom) off-centered positioning. The reconstructions were performed offline from the average of 10 repeated projection datasets to mitigate the effect of measurement noise. The same narrow grayscale window is used for both images, namely $\{\text{Level, Width}\} = \{0,80\}$ HU.

The ACR phantom was scanned in two different positions: centered, and off-centered by a vertical shift of 4.8 cm up. For off-centered positioning of the phantom, image reconstruction was performed on a shifted grid of pixels that exactly compensated for the vertical shift. The images obtained offline after averaging of the repeated scans are shown in figure 5. This figure shows that the scanning plane cuts through the phantom in a closely similar manner for both positions of the phantoms: the four steel beads at the cardinal points are similarly visible, and the number of visible wires from the ramps remains the same from one position to the other. Another aspect to observe in these images is the beam hardening errors associated with the bone insert (upper right quadrant). Not surprisingly, these errors are more pronounced in the LE results. Also, in both LE and HE cases, they are more pronounced and asymmetrical when the phantom is off-centered. Such differences due to positioning were expected and they motivated our decision to investigate accuracy for two positions of the phantom; since the energy response is dependent on the detector channel, off-centered positioning of the phantom breaks all symmetries and represent a more challenging experiment than centered positioning. Another motivation is that patient scans are rarely centered, and 4.8 cm is fairly representative of clinical situations. Nevertheless, centered positioning is interesting as well since this is how the ACR phantom is usually scanned.

### 3.4. Evaluation methodology

First, some technical aspects of our data-based material decomposition were assessed. Specifically, we evaluated how the parameters involved in our scatter subtraction scheme (figure 2) varied with phantom position and with the scan energy (i.e. LE versus HE). Also, we evaluated the number of iterations needed for application of this scheme in each of these four settings.

Next, image quality was assessed both visually and quantitatively. Visual assessment was focused on inspection of monochromatic images in terms of noise and artifacts; two energies were considered, namely the common energy of 70 keV, and a lower energy of 50 keV, which can be of clinical value to improve contrast.

All quantitative assessments were made across the 30 to 140 keV energy range, using six regions of interests (ROIs) that are depicted in figure 4. Five of these ROIs were circular regions covering the central portion of the five inserts with a fixed diameter of 20 mm; these are denoted as ROIs $\#1$ through $\#5$ in figure 4. Using a diameter smaller than that of the insert was chosen to prevent interference with resolution effects at the edge of the inserts. The sixth ROI was a ring-shaped region drawn around the bone insert to assess the magnitude of the beam hardening errors where they are expected to be strongest; this ROI is delimitated by two green circles in figure 4. The thickness of the ring was 4.9 mm, and its inner radius, measured from the center of the bone insert, was 17.64 mm.

We performed an evaluation of noise in terms of standard deviation within the water insert (namely, ROI $\#5$ in figure 4). Afterwards, we performed evaluations of accuracy using mean values over all five circular ROIs (namely, ROI $\#1$ through $\#5$ in figure 4) plotted on both absolute and relative scales. And we performed evaluation of strength in beam hardening error using the ring-shaped ROI using the mean absolute pixel difference from the GT, to avoid cancellation of errors.

## 4. Additional experiments

In addition to the experiments detailed above, which were focused on assessing accuracy in the reconstructed attenuated values, we conducted a small subset of other experiments with the following aims regarding the proposed method: (1) to assess the impact of scatter subtraction, (2) to assess performance over conventional data-based material decomposition, (3) to assess performance in an imaging scenario that is closer to clinical practice. Specific details for each of these aims are given below.

### 4.1. Impact of scatter subtraction

In our previous work on water-based beam hardening correction, we already showed that scatter subtraction is important for accuracy (Haase *et al* 2022). We repeated such experiments, now in the context of DE imaging. Specifically, we performed data-based material decomposition using our method but without applying the scatter subtraction scheme of figure 2. This amounts to immediately applying the approach of figure 3 with content of the input box replaced by ‘projection data of LE and HE scans before vendor correction’.

For the assessment here, we only used the CT data of the ACR phantom scanned in the centered position. The impact of scatter subtraction was visually assessed through inspection of image differences, which de-emphasizes quantum noise and thereby allows better visualization of scatter contributions. We also performed some quantitative assessment using a thick vertical profile through the difference images. This profile was drawn through the polyethylene and acrylic inserts with a thickness of 16 mm, which roughly covers 2/3 of the inserts diameter.

### 4.2. Comparison with conventional data-based material decomposition

Data-based material decomposition has been conventionally described as follows. First, use a calibration phantom to identify the mathematical relationship between the measurements and lengths of two materials. Second, invert this relationship to reconstruct the density distribution of these two materials within the object. Last, create mono-energetic images through recombination. For computational simplicity, the inverse relationship can be directly modeled as polynomials that transform the measurements into lengths of the two materials, as suggested in Lehmann *et al* (1981).

Since our implementation of the image-based material decomposition involved collecting HE and LE data of an expensive calibration phantom, it is natural to ask if similar data could be used to implement this conventional data-based material decomposition and how good the results would be, compared to the proposed approach. To address this question, we collected HE and LE scans of the multi-energy CT phantom using one calcium insert successively placed at two locations: first, at the center, as shown in figure 1 (right); second, at the 9 o’clock position. The images obtained from the HE scans were segmented into two masks, one that covered the calcium inserts, and one that covered the water-equivalent material. Then, fan-beam forward projection of the four masks was used to create a comprehensive set of water and calcium lengths that could be used for polynomial fitting.

Polynomial fitting to transform the DE measurements into lengths of the two materials was done through least-square minimization and disregarded variations in energy response from one detector pixel to another. While fitting one polynomial per detector pixel would have allowed accounting for variations in energy spectrum, such fitting could not be stably performed because the range of material lengths covered for each detector pixel was too small. Like in the image-based approach, scatter was somewhat accounted for as the calibration data (and thus the fitted polynomials) included scatter signal from the calibration phantom. Two polynomial fitting options were considered. The first option was cubic; specifically, we used

\begin{eqnarray*} L_{\text w} &amp; = \sum_{1\unicode{x2A7D} k+l\unicode{x2A7D} 3,\, k\unicode{x2A7E} 0,\, l\unicode{x2A7E} 0} a_{k,l}^{\text w} \, g_{\mathrm{HE}}^k \, g_{\mathrm{LE}}^l\,,\end{eqnarray*}

\begin{eqnarray*} L_{\text c} &amp; = \sum_{1\unicode{x2A7D} k+l\unicode{x2A7D} 3,\, k\unicode{x2A7E} 0,\, l\unicode{x2A7E} 0} a_{k,l}^{\text c} \, g_{\mathrm{HE}}^k \, g_{\mathrm{LE}}^l\end{eqnarray*}

where *g*_HE_ and *g*_LE_ are the high and low energy measurements obtained at any detector pixel, $a_{k,l}^{\text w}$ and $a_{k,l}^{\text c}$ are the fitted coefficients, and $L_{\text w}$ and $L_{\text c}$ are the estimated lengths of water and calcium. The second option was of higher order (namely quartic) while using a more constraining form:

\begin{eqnarray*} L_{\text w} &amp; = b_1^{\text w} \, \left(g_{\mathrm{HE}}+g_{\mathrm{LE}}\right) + \sum_{k = 2}^{4} b_k^{\text w} \, \left(g_{\mathrm{HE}}-g_{\mathrm{LE}}\right)^k\end{eqnarray*}

\begin{eqnarray*} L_{\text c} &amp; = b_1^{\text c} \, \left(g_{\mathrm{HE}}-g_{\mathrm{LE}}\right) + \sum_{k = 2}^{4} b_k^{\text c} \, \left(g_{\mathrm{HE}}-g_{\mathrm{LE}}\right)^k\end{eqnarray*}

where $b_k^{\text w}$ and $b_k^{\text c}$ are the fitted coefficients.

For comparison between the proposed approach and this conventional data-based material decomposition, we again used the CT data of the ACR phantom scanned in the centered position. We limited the comparison to visual side-by-side assessment of mono-energetic images obtained at 70 keV. For completeness and fairness, we also created a version of our approach that treats all detector pixels as the central one, thus neglecting the effect of the bowtie filter on the energy response.

### 4.3. Testing with an anthropomorphic phantom

To assess performance of the proposed approach in a scenario that is closer to clinical practice, we acquired HE and LE scans of an abdomen phantom (QRM, Möhrendorf, Germany). The phantom included an extension ring making its in-plane dimension equal to 40 cm × 30 cm. The scanning parameters were the same as in table 1, except for the tube current which was 240 mAs for the HE scan and 600 mAs for the LE scan. As before, to mitigate quantum noise, all scans were acquired 10 times and averaged together before image reconstruction. Full-scan reconstructions were performed with a slice thickness of 6 mm and a smooth convolution kernel designed for quantitative applications that is called ‘D20s’. The field-of-view was a circle of diameter equal to 500 mm, leading to a pixel size of 0.98 mm.

Since chemical composition of the phantom components was not known, accuracy in the reconstructed attenuation values could not be assessed. Instead, the evaluation was focused on assessing consistency in the attenuation values that is achieved when scanning the phantom at two different positions: centered and off-centered by a vertical shift of 40 mm up. Visual assessment was performed as well as quantitative assessment using a 40 mm thick vertical profile.

## 5. Results on accuracy of attenuation values

### 5.1. Technical aspects

Analysis of the calibration scans used for material decomposition in the image domain produced relative density values of 1.0053 and 2.1429 for water and bone at the LE setting, which resulted in a ratio of 2.1316 leading to an effective energy $\varepsilon_{\mathrm{LE}} = 64.0$ keV for the LE scan. For the HE scan, relative density values of 1.0035 and 1.8446 were obtained for water and bone, which resulted in a ratio of 1.8382 leading to an effective energy $\varepsilon_{\mathrm{HE}} = 78.5$ keV. Note that the relative density values reported here vary with tube voltage as they are expressed relative to an energy-independent attenuation value for water.

The optimization scheme for scatter subtraction, described in figure 2, was applied with a scatter kernel width, *σ*, of 5 detector pixels. The scheme produced values for two parameters, *α* and *β*, which are reported in table 2 for the central detector row, for both positions of the phantom and both energy settings. See Haase *et al* (2022) for mathematical details regarding how *α*, *β*, and *σ* are used to define the scatter profile. The value of *σ* was not investigated; instead, we used the value that was empirically found to be suitable in Haase *et al* (2022). The iterations of the simplex algorithm were initiated with $\alpha = \beta = 1$ and stopped when the change in the cost function and the changes in *α* and *β* were less than 10^−4^. The number of iterations that were needed are also reported in table 2. As can be observed, the values of *α*, *β*, and the number of iterations were relatively similar for both positions of the phantom.

**Table 2.** Scatter parameter results of optimization scheme for scatter subtraction. Presented values for *α*, *β*, and number of iterations needed for convergence of the optimization scheme are given for the central detector row.

Input projection data	*α*	*β*	iterations
Centered position (HE)	2.6044	0.9554	58
Centered position (LE)	1.9627	0.9421	46
Off-centered position (HE)	2.4211	0.9386	53
Off-centered position (LE)	1.8843	0.9365	52

The second step of the data-based material decomposition, described in figure 3, required specifying intervals of material lengths covered by polynomials. For water, we used lengths spanning the interval $[-2.0, 28.0]$ cm with a 0.1 cm step. For calcium, we used lengths spanning the interval $[-4.0, 5.0]$ cm also with a 0.1 cm step. The estimated material lengths were found to fit within these intervals. Quintic polynomials were found to be the lowest order polynomials providing an adequate fitting (again, see Haase *et al* (2023) for further details on this aspect).

### 5.2. Visual and quantitative aspects

The results obtained for the two positions of the phantom are shown together in each figure; the top row shows the results from centered positioning and the bottom row shows results from off-centered positioning. This arrangement allows individual assessments while facilitating a visual appreciation of differences between the two cases, as already done in figure 5.

**Figure 6.**
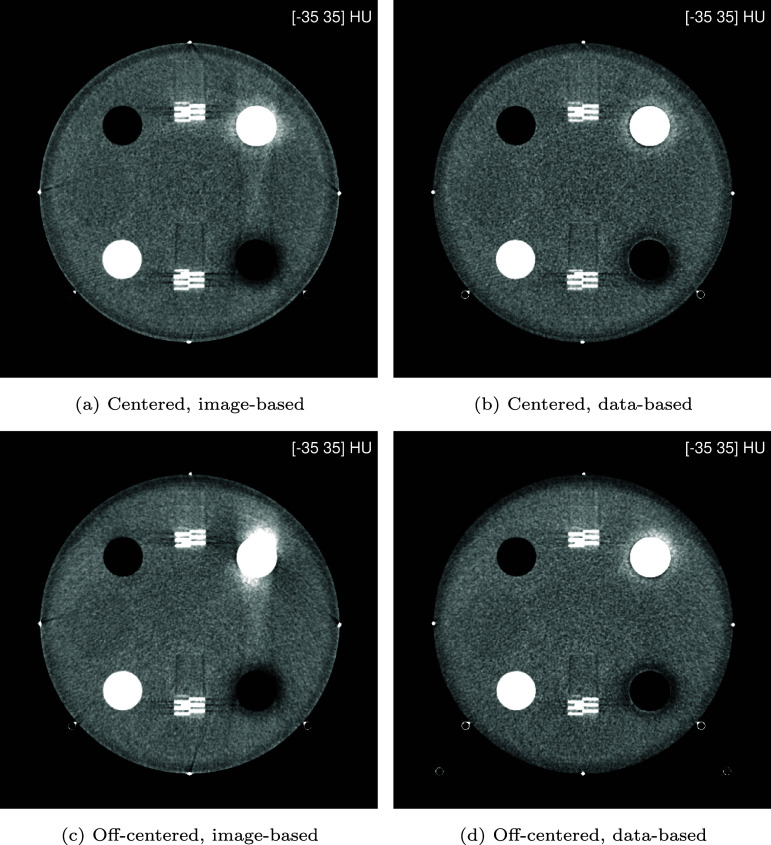
Virtual mono-energetic images at 70 keV for centered and off-centered positioning of the phantom: (left) image-based and (right) data-based material decomposition.

**Figure 7.**
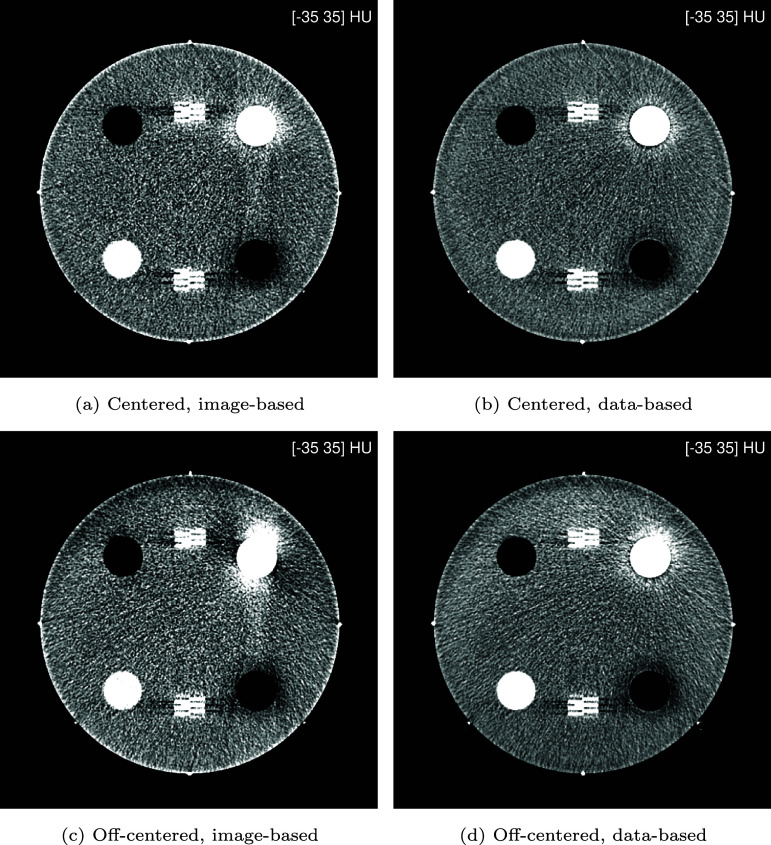
Virtual mono-energetic images at 50 keV for centered and off-centered positioning of the phantom: (left) image-based and (right) data-based material decomposition.

We begin the presentation of results with a visual inspection of virtual mono-energetic images. Figure 6 shows such images at 70 keV, which is an energy often used in clinical practice. Then, figure 7 shows such images at the lower energy of 50 keV, which is an energy that can be useful for specialized clinical applications. In both centered and off-centered positioning, similar differences are observed between the image-based and data-based material decomposition approaches. First, the image-based approach largely retains beam hardening artifacts seen in the original, polyenergetic, LE and HE images (figure 5), particularly around the bone insert and between the bone and air inserts. Second, images at 50 keV are generally noisier, with the image-based approach exhibiting a more pronounced noise increase. Third, streak artifacts originating from the metal beads appear in the image-based results but are absent in the data-based reconstructions. Overall, the data-based approach yields superior visual image quality in both phantom positions. Nevertheless, some limitations remain: low-frequency (halo-like) artifacts persist around the bone and air inserts, most visible at the 50 keV level, and these are less radially uniform in the off-centered position; and slight undershoot/overshoot can be observed at the edges of these inserts.

**Figure 8.**
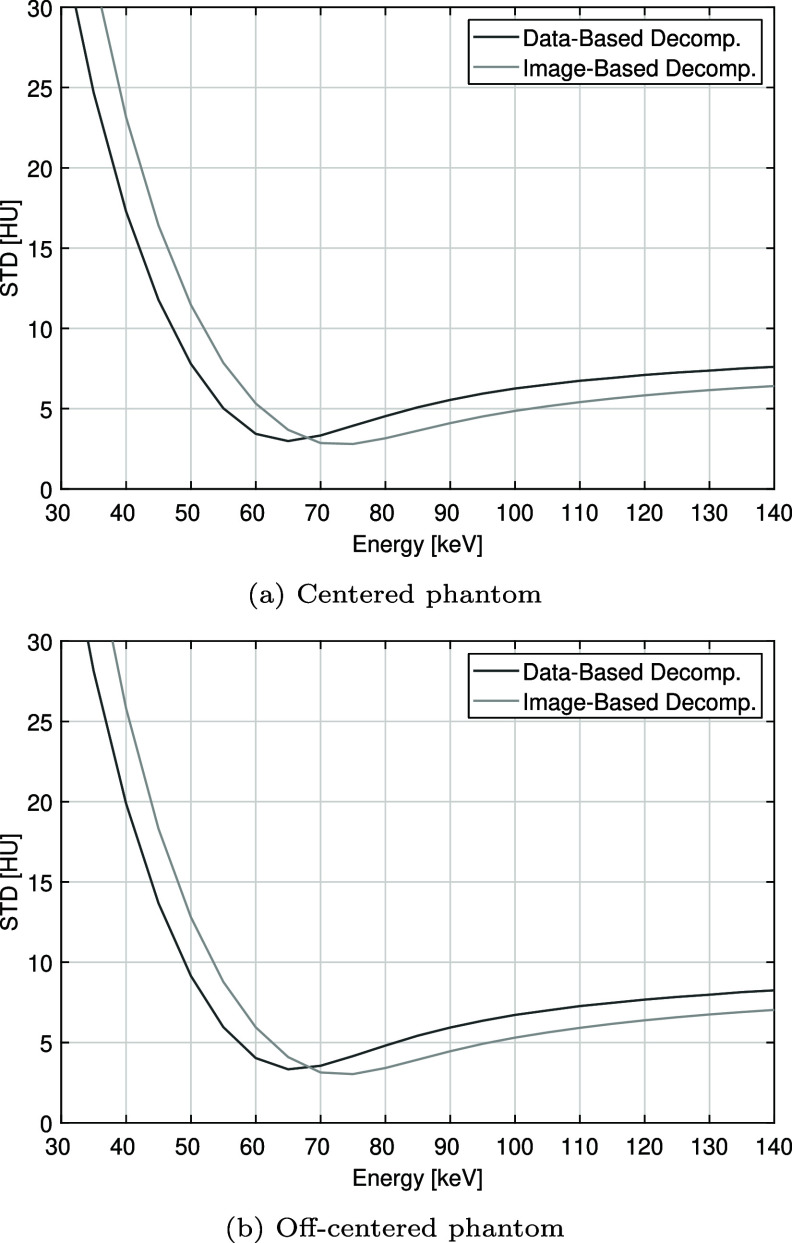
Standard deviation (STD) over the water insert (ROI $\#5$ in figure 4) as a function of energy using image-based and data-based material decomposition. (Top) centered phantom: for the image-based approach, the minimum is 2.80 HU, reached at 75 keV; for the data-based approach, the minimum is 2.98 HU, reached at 65 keV. (Bottom) off-centered phantom: for the image-based approach, the minimum is 3.03 HU, reached at 75 keV; for the data-based approach, the minimum is 3.33 HU, reached at 65 keV.

Now, the results are presented in a quantitative manner, starting with an analysis of noise behavior as a function of the energy level used for the mono-energetic images. Figure 8 shows that both material decomposition approaches exhibit a parabolic noise profile, with noise rapidly increasing below a characteristic energy minimum, and this behavior is not affected by phantom positioning. However, and importantly, the data-based approach consistently reaches its noise minimum at an energy approximately 10 keV lower than the image-based approach. This observation confirms the visual impression of lower noise at 50 keV for the data-based approach, seen in figures 6 and 7.

**Figure 9.**
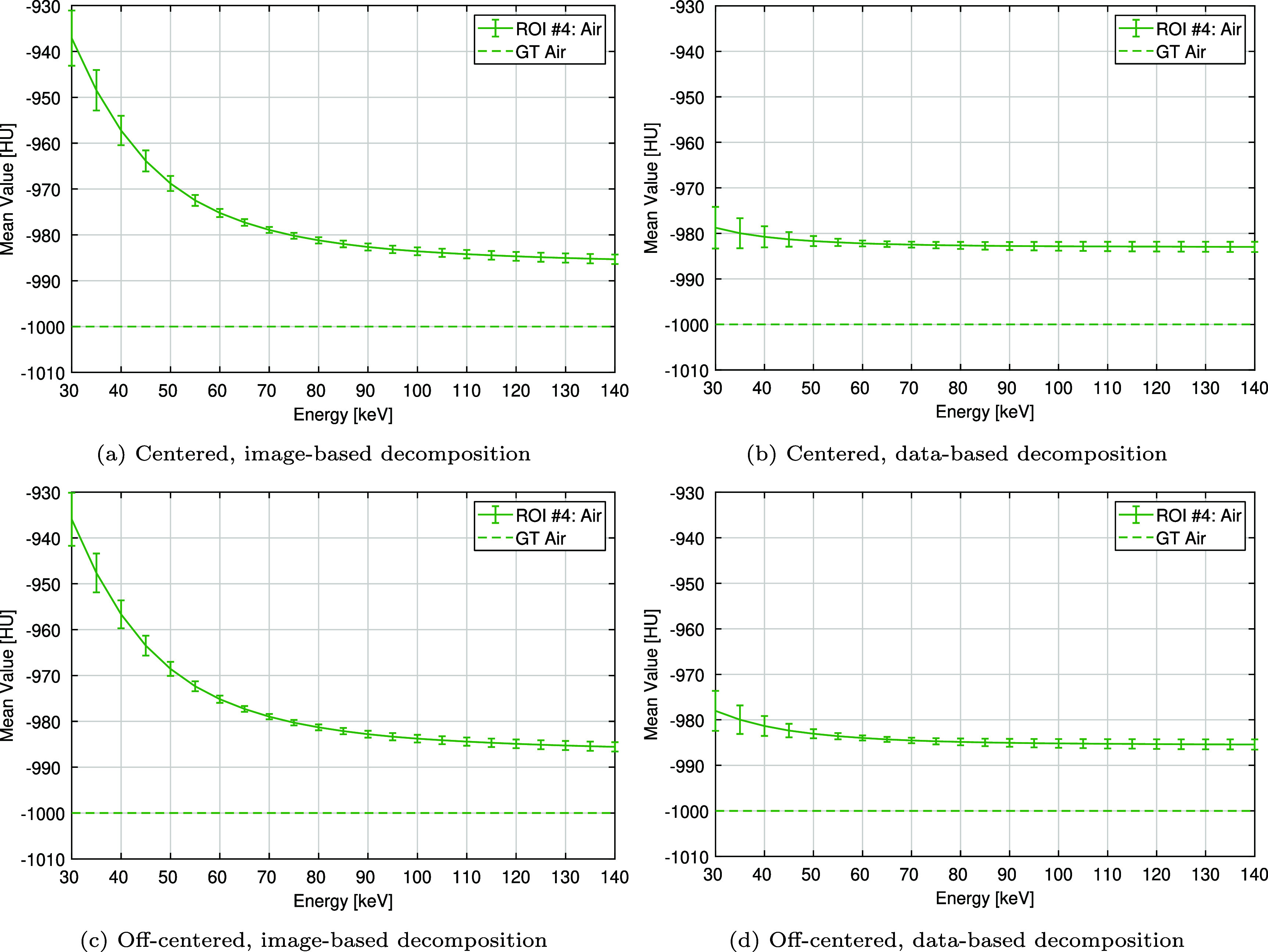
Mean value within the air insert (ROI $\#4$ in figure 4) as a function of energy. (Top row) centered phantom. (Bottom row) off-centered phantom. (Left) with image-based material decomposition. (Right) with data-based material decomposition. The error bar corresponds to ±1 standard deviation, estimated from the scan repetitions. Here,‘GT’ stands for ground truth. Notice how accuracy deteriorates much faster for the image-based approach than the data-based approach at energies below 70 keV.

Figure 9 shows the accuracy of Hounsfield unit (HU) values within the air insert (ROI $\#4$) across the 30 to 140 keV energy range. For both phantom positions, the data-based decomposition shows a more uniform trend, with mean values decreasing from −979 HU to −983 HU (centered) and from −978 HU to −985 HU (off-centered). In contrast, the image-based decomposition exhibits greater variability, ranging from −938 HU to −986 HU (centered) and −936 HU to −986 HU (off-centered). Moreover, at low energies, the image-based approach shows a much more pronounced degradation in accuracy as a function of the energy. E.g., at 50 keV and 70 keV, the slope, expressed in HU/keV, is −0.87 and −0.29 for the image-based approach compared to only −0.09 and −0.03 for the data-based approach (calculated as an average over centered and off-centered positions). At 140 keV, the image-based result is marginally closer to the expected value of −1000 HU, by 3 HU and 1 HU in the centered and off-centered positions, respectively. However, this expected value is never reached: the smallest absolute error across positions and material decomposition methods is 14 HU at 140 keV.

**Figure 10.**
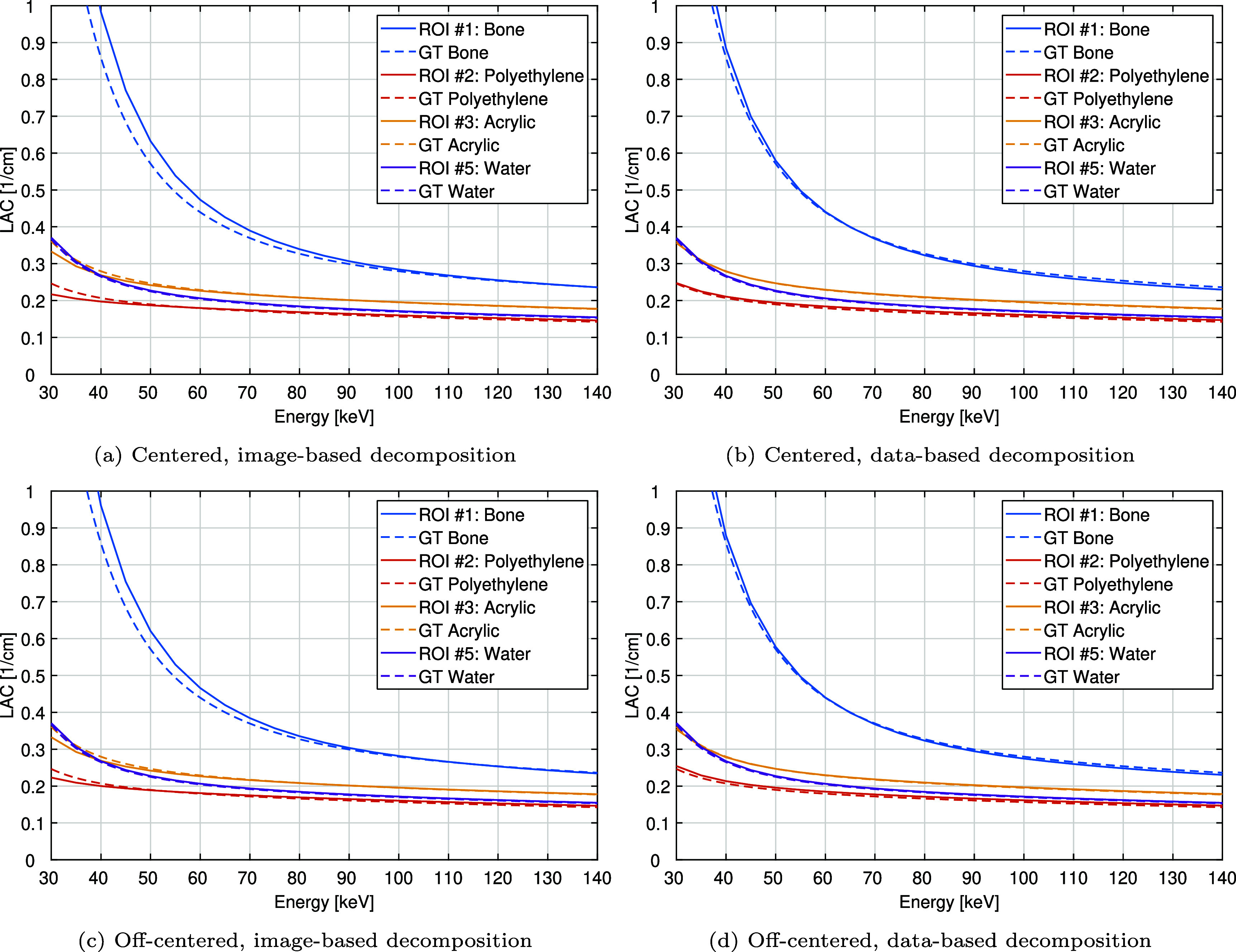
Mean value within the bone, polyethylene, acrylic, and water inserts (ROIs $\#1$, $\#2$, $\#3$, and $\#5$ in figure 4) on an absolute scale as a function of energy, and as obtained from the reconstructed mono-energetic images of the phantom in the centered and off-centered positions: (left) image-based and (right) data-based material decomposition. Here, ‘GT’ stands for ground truth.

**Figure 11.**
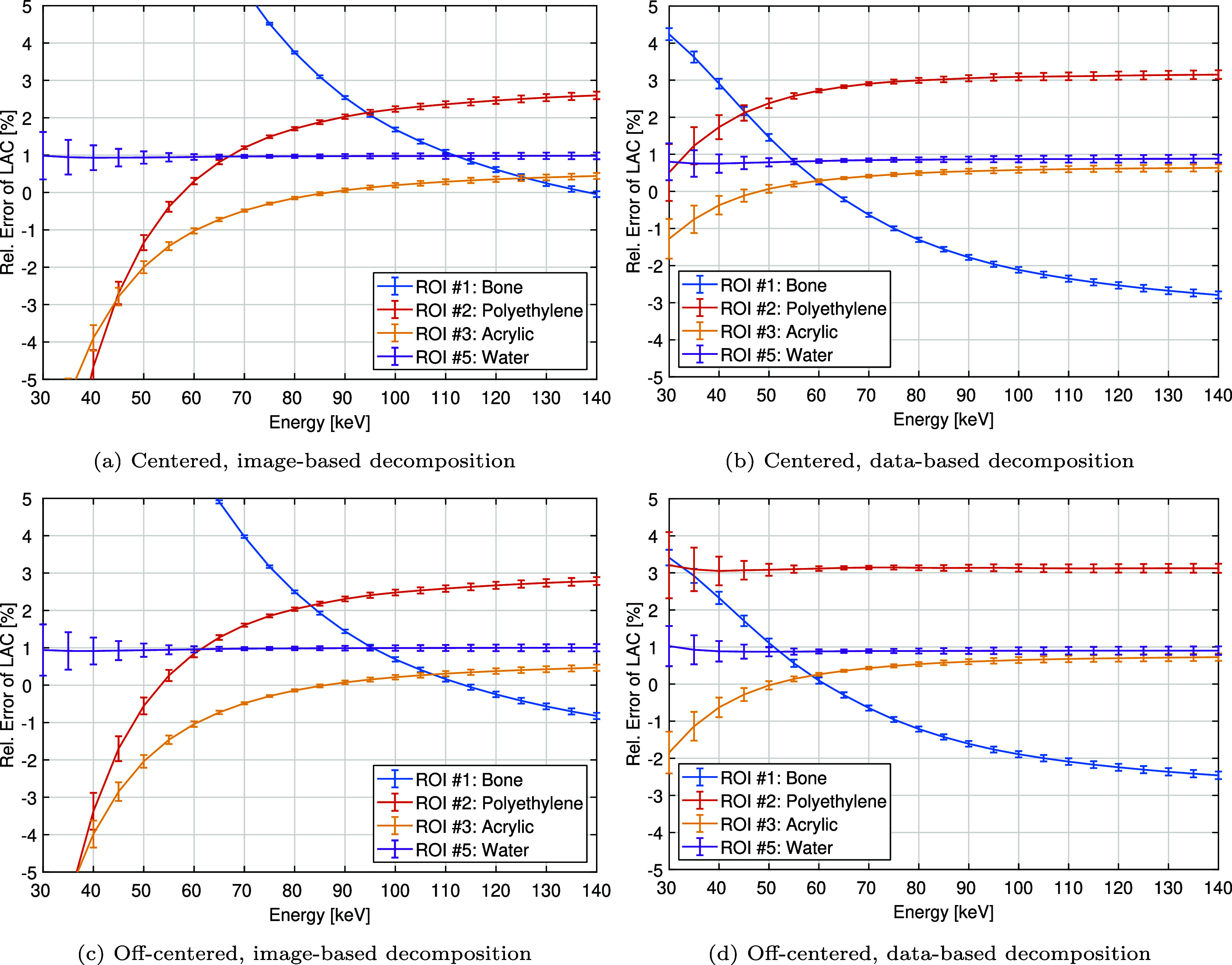
Mean value within the bone, polyethylene, acrylic, and water inserts (ROIs $\#1$, $\#2$, $\#3$, and $\#5$ in figure 4) on a relative scale as a function of energy, and as obtained from the reconstructed mono-energetic images of the phantom in the centered and off-centered positions: (left) image-based and (right) data-based material decomposition. The error bar corresponds to ±1 standard deviation, estimated from the scan repetitions.

Figures 10 and 11 show the accuracy of HU values within the bone, polyethylene, acrylic, and water inserts (ROIs $\#1, \#2, \#3$, and $\#5$, respectively) across the 30 to 140 keV energy range. Accuracy is shown on both absolute scale (figure 10) and relative scale (figure 11): absolute values are compared to GT with dashed curves; and relative errors, computed relative to the GT values, are visualized with error bars indicating ±1 standard deviation from scan repetitions. On the absolute scale, all materials except bone align well with GT, while bone exhibits larger deviations, especially in the image-based decomposition at energies below 70 keV. Relative error analysis provides finer details. Water is consistently reconstructed with high accuracy (absolute relative error below 1%) across all energies and both methods. For bone, polyethylene, and acrylic, the data-based decomposition demonstrates more uniform and generally smaller relative errors compared to the image-based method. Specifically, bone errors range from 18.2% to −0.1% (centered) and 15.2% to −0.8% (off-centered) for image-based, and from 4.2% to −2.8% (centered) and 3.4% to −2.5% (off-centered) for data-based decomposition. Polyethylene errors span −11.9% to 2.6% (centered) and −9.5% to 2.8% (off-centered) for image-based, compared to 0.5% to 3.1% (centered) and 3.2% to 3.1% (off-centered) for data-based decomposition. Acrylic errors range from −7.7% to 0.4% (centered) and −7.9% to 0.5% (off-centered) for image-based, versus −1.3% to −0.6% (centered) and −1.8% to 0.7% (off-centered) for data-based decomposition. Moreover, as shown in table 3, at low energies, the image-based approach shows a much more pronounced change in accuracy with energy, similar to what was observed in the air insert.

**Table 3.** Energy-dependence of the rate of change in the relative error in mean value within the bone, polyethylene, and acrylic inserts shown in figure 11. Each absolute value of the slope is reported as an average over centered and off-centered positions and is expressed in $\%$ keV.

	Absolute value of slope at		
	50 keV	70 keV	90 keV
ROI $\#1$: Bone			
Image-based	0.32	0.19	0.10
Data-based	0.13	0.07	0.04
ROI $\#2$: Polyethylene			
Image-based	0.21	0.06	0.02
Data-based	0.02	0.01	0.00
ROI $\#3$: Acrylic			
Image-based	0.14	0.04	0.02
Data-based	0.04	0.01	0.00

**Figure 12.**
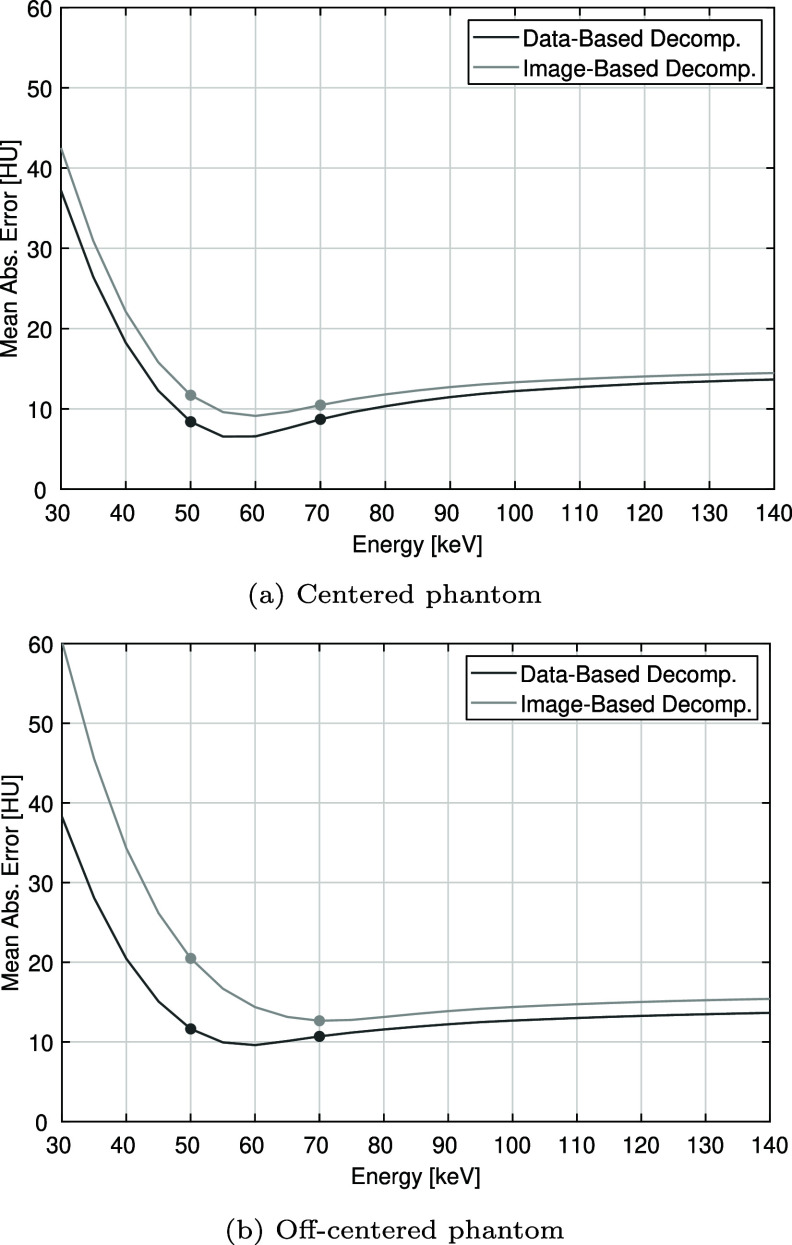
Mean absolute relative error inside the ring ROI towards the ground truth material of the phantom. (Top) centered phantom: for image-based decomposition, the values at 50 keV and 70 keV are 11.7 HU and 10.5 HU, respectively; for data-based decomposition, these values are 8.4 HU and 8.7 HU. (Bottom) off-centered phantom: for image-based decomposition, the values at 50 keV and 70 keV are 20.5 HU and 12.6 HU; for data-based decomposition, they are 11.6 HU and 10.7 HU.

Last, figure 12 presents the mean absolute error around the bone insert (using the ring-shaped ROI). While not providing perfect results as previously observed in figures 6 and 7, the data-based method clearly yields lower errors throughout the 30 to 140 keV range, in both phantom positions, and most particularly for the off-centered position. Independently of the method and the phantom position, the error always follows a similar pattern: sharply decreasing towards a minimum, then slowly increasing as the energy is further increased. For the centered position, the minimum errors are 6.5 HU at 55 keV (data-based) and 9.1 HU at 60 keV (image-based). In the off-centered position, these minima shift to 9.6 HU at 60 keV and 12.6 HU at 70 keV, respectively. The maximum errors are reached at 30 keV, and they are 37.2 HU and 42.5 HU for the centered case (data-based and image-based, respectively), and 38.3 HU and 60.2 HU for the off-centered case. Note that in this figure, we also marked the data points that correspond to the mono-energetic images shown in figures 6 and 7.

## 6. Results of additional experiments

**Figure 13.**
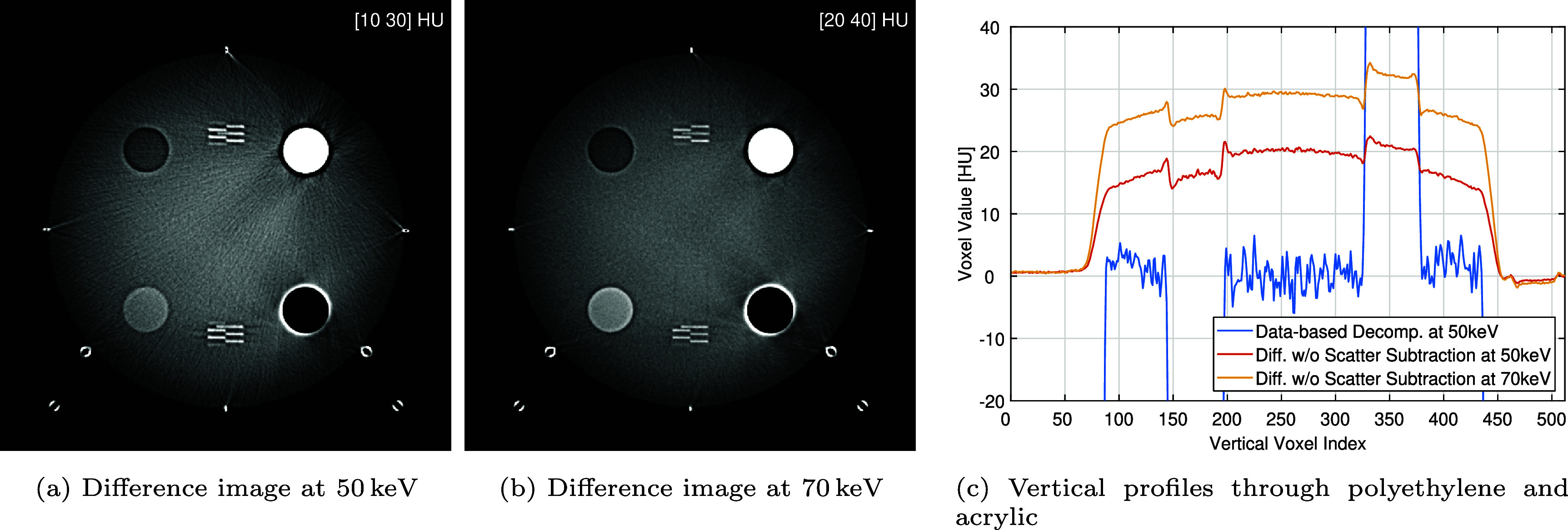
Effect of scatter correction in the proposed data-based decomposition: (a) and (b) show the difference in the virtual mono-energetic images at 50 keV and 70 keV that result from removing the scatter correction step, with grayscale windows specified in the upper right corner; (c) 16 mm-thick profiles through the difference images shown in (a) and (b) as well as through the 50 keV virtual mono-energetic image obtained with the proposed approach, drawn along the vertical line that passes through the center of the polyethylene and acrylic inserts.

The results on the impact of scatter subtraction are shown in figure 13. As can be seen in the difference images, disregarding scatter leads to important errors in the form of shading, cupping and streaks artifacts. The magnitude of the error can be as high as 34 HU. Also, this magnitude depends on the energy level in a somewhat unpredictable manner as the result at 50 keV is more accurate by 10 HU than the result at 70 keV.

**Figure 14.**
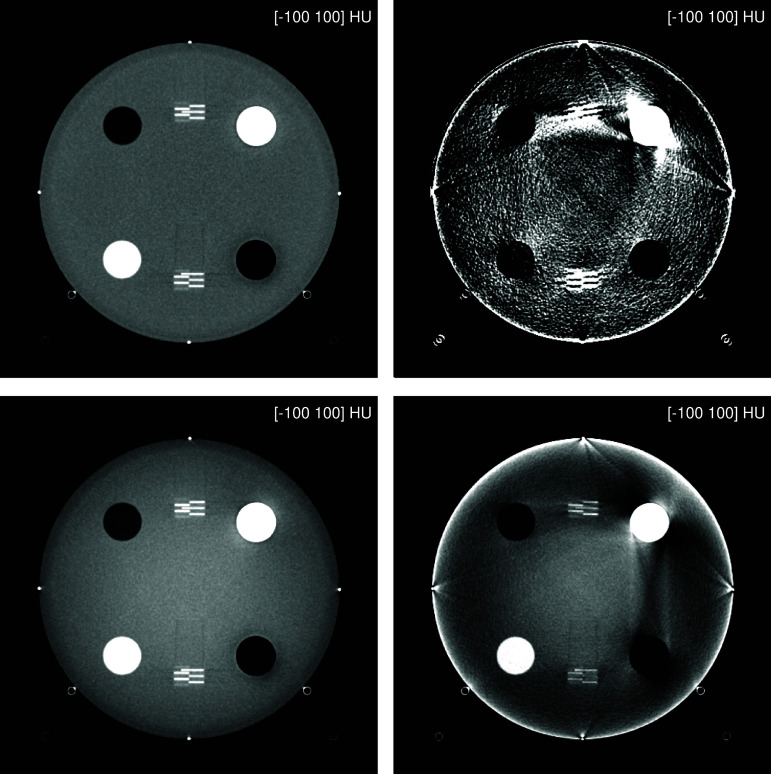
Comparison between the proposed approach and conventional data-based material decomposition. Virtual mono-energetic images at 70 keV are shown for the centered phantom. Top left: proposed approach. Bottom left: simplified version of the proposed approach where the energy spectrum is assumed to be the same for each detector pixel as that of the central detector pixel. Top right: conventional approach using cubic polynomials. Bottom right: conventional approach using constrained quartic polynomials. See the text for the mathematical expression of the polynomials. Note that a wider grayscale window is used in this figure, compared to figure 6.

The 70 keV results on comparative utilization of conventional data-based material decomposition are displayed in figure 14. Note that a wider grayscale window had to be used to display these results (compared to figure 6). As can be seen, the conventional data-based approach produces strongly unsatisfactory results, even when compared with the simplified version of the proposed approach that disregards variations in energy response across the detector pixels. There are strong streak artifacts linking the bone insert to be the metal beads at the cardinal points of the phantom. There are also strong cupping and shading artifacts throughout the phantom. The second polynomial option, however, does provide results with less prominent artifacts and less noise than the first option.

**Figure 15.**
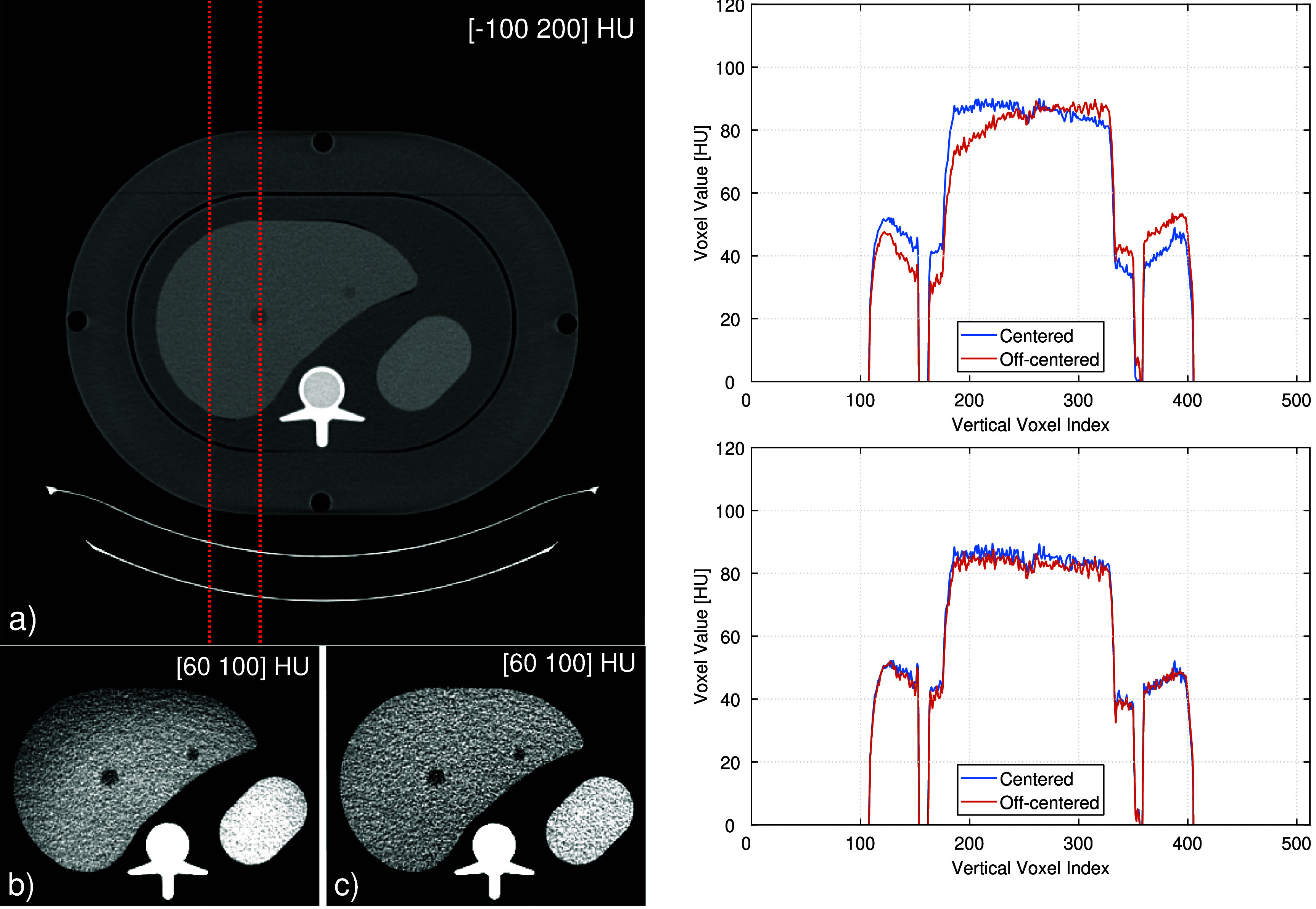
Experimental results obtained at 80 keV using the QRM abdomen phantom with fat belt. The images on the left were obtained using: (a) the proposed method with phantom centered on the rotation axis; (b) the image-based material decomposition with the phantom off-centered by 4 cm in the vertical direction; (c) the proposed method with the same off-centered phantom positioning as in (b). The grayscale windows are specified in the upper right corner of each image. The plots on the right show a thick vertical profile at the location identified by the two dotted red lines in (a). The top plot is for the image-based decomposition, while the bottom plot is for the proposed method. Each plot includes two profiles that correspond to the phantom being centered or off-centered; ideally, the two profiles should overlap.

The results with the QRM abdomen phantom are displayed in figure 15. The most salient points to observe here are as follows. First, when the phantom is off-centered, the image-based approach yields a very non-uniform attenuation throughout the liver compared to the proposed approach. Second, for the image-based approach, the change of 40 mm in vertical position of the phantom causes non-local and non-uniform variations in the attenuation values that can be as high as 16 HU, whereas the proposed approach yields much more similar attenuation values across the two phantom positions (differences on the order of 2 to 6 HU in the shown profile).

## 7. Discussion

### 7.1. Aspects of accuracy experiments

We presented results from two experiments, one using the ACR phantom centered on the rotation axis of the scanner, and the other one using the same phantom vertically shifted by 4.8 cm up, which is a technically more challenging imaging scenario. Our evaluation of the results was primarily focused on image accuracy. These results showed relatively important advantages for the data-based decomposition approach proposed in this work, in comparison with the image-based approach. These advantages may be summarized as follows.

First, within the body of the phantom but outside the inserts, where beam hardening errors are most obvious, we observed much improvements in that the pattern between the bone and air inserts disappeared, and the low-frequency (halo-like) errors around these inserts were much reduced although not fully eliminated. Since there should be no beam hardening errors around the air insert, we suspect that data aliasing from sharp contrast within the image reconstruction process may be partly responsible for the remaining errors around the bone and air inserts, but further investigation is needed to clarify this issue. An alternative could be that scatter is not suitably well corrected for. In addition, we observed that the data-based approach avoided streaks from the metal beads at the cardinal points of the phantom, which may be important for clinical problems were metal wires or calcium deposits hamper clinical interpretation.

Second, we observed that the data-based approach provided much higher accuracy within the bone insert at energies below 70 keV while performing similarly at higher energies. Within the acrylic and polyethylene inserts, both methods performed quite well, but the data-based approach yield more uniform accuracy across the whole 30 to 140 keV energy range, while the image-based result exhibit strong divergence below 40 keV. This might explain why most vendors limit the lowest energy for mono-energetic images to 40 keV. The much higher accuracy of the data-based approach at 30 keV could potentially be beneficial for some clinical applications, particularly as some studies have already showed values of 40 keV images (such as Noda *et al* (2020) for pancreatic cancer and Lam *et al* (2015) for head and neck tumors).

Within the air insert, we observed that the expected value of −1000 HU was not reached with either method, with the image-based approach marginally better at high keV, while the data-based approach showed a fairly uniform trend across the 30 to 140 keV energy range. The fact that −1000 HU is not reached could again be due to data aliasing during image reconstruction or due to remaining scatter effects.

While the data-based approach clearly produced more accurate results towards the low energies, it should be kept in mind that noise quickly increases as the energy is decreased. In this regard, the parabolic noise behavior that we observed with both approaches was consistent with other reports; see, e.g. Yu *et al* (2012) for a review on this topic, and see Alvarez and Seppi (1979) for an explanation based on data statistics. However, it is clinically valuable that the data-based approach reaches its minimum in noise standard deviation at an energy that is 10 keV lower.

### 7.2. Aspects of additional experiments

The additional experiments showed the importance of accounting for scatter in the proposed approach as well as the importance of accounting for variations in the energy response across the detector pixels (which are induced by the bowtie filter). They showed that the proposed approach also outperforms the image-based approach in a scenario closer to clinical practice, namely abdominal CT imaging. Last, they showed that the proposed approach strongly outperforms conventional data-based material decomposition when the latter is implemented using the same calibration phantom as the image-based approach.

This last aspect is not surprising. The shown results primarily convey the complexity of implementing conventional data-based material decomposition. An important challenge lies in the difficulty of including a wide range of water and calcium lengths to achieve a robust polynomial fitting. While our implementation covers the whole range of lengths seen in the ACR phantom, small lengths were actually poorly covered due to the rapid change in the length of intersection between circles and lines. In the first polynomial fitting, the first order coefficients for the water lengths were unexpectedly large and of opposite sign, which led to high noise. The second polynomial fitting avoided this issue. To the best of our knowledge, there exists no standardized calibration phantom that covers well a wide range of material lengths, and the issue becomes even more challenging when individual polynomial fittings are desired for each detector pixel.

### 7.3. General aspects and limitations

Using the data-based approach, one finds that the relative error within the solid inserts of the ACR phantom varies between ±4 $\%$, which may or may not be seen as impressive. In this aspect, one should realize that the achievable accuracy is not limited only by data processing. First, the decomposition using two basis materials is only an approximation, albeit a good one. For example, our results tend to convey that acrylic is better represented by our two basis functions than polyethylene. Second, the accuracy of our GT is influenced by how well the atomic composition and mass densities of our instance of the ACR phantom match those specified by the manufacturer. High fidelity can be expected since the ACR phantom is manufactured with high quality control standards, but there is the side aspect that the mass densities of the phantom components are only provided with two decimals (in g cm^−3^), which limits the precision with which the GT is known. An alternative to using the ACR phantom for our evaluation experiments could have been to use the Multi-Energy CT phantom (Sun Nuclear, Melbourne, FL, USA), which may offer higher precision as the mass densities for this phantom are given to three decimal places. The ACR phantom was ultimately chosen for our evaluation experiments because the Multi-Energy CT phantom was prioritized for calibrating the image-based approach and we did not want to create a bias towards the image-based approach by using the same phantom for both calibration and evaluation.

Another reason to select the ACR phantom for our evaluation experiments is the familiarity of medical physicists with the ACR phantom as this phantom is widely used for quality control. Nevertheless, some medical physicists may possibly find our results confusing, in that they may not be familiar with seeing the artifacts we have shown. This is due to us using a narrower grayscale window than used for quality control, and also due to considering of an off-centered position for the phantom. One of the co-authors verified that CT imaging at another institution (University of California in Los Angeles, USA) using scanners from the same vendor leads to similar artifacts as those shown in figure 5.

The evaluation required making a choice for the energy of the scans. The decision of using scans collected at 80 kV and 120 kV is somewhat unconventional, since nowadays most x-ray tubes can acquire data at 140 kV or even higher. Our reasoning in this aspect was that we wanted to create a more challenging scenario. Using 140 kV or more for the HE scan is preferred in clinical practice, as it reduces sensitivity to noise. However, our investigation was focused on accuracy, not noise. The issue of noise was controlled using an average of repeated scans, which allowed us to tackle a more challenging DE material decomposition scenario.

The beam hardening errors in the image-based approach may not be easy to mitigate: as we have seen, they largely originate from the low energy scan, which is critically needed to perform stable material decomposition. Also, the predictability of beam hardening errors may be worse for larger objects, as the shift-variant energetic effect due to the bowtie filter starts playing a larger role. As implemented, the image-based approach uses a single calibration matrix based on measurements near the center of the gantry, where the bowtie filter is locally shift-invariant. Position-dependent calibration matrices may be useful, but have not been investigated and are not, to our knowledge, used in practice.

While our experimental results support the data-based approach, the evaluation did not account for patient motion. Data-based material decomposition is known to be sensitive to patient motion, and using consecutive scans to obtain the LE and HE measurements increases risk of patient motion. Hence, direct clinical application of our results lies most in imaging scenarios where motion can be well controlled. Contrast-free consecutive spiral scans for kidney stones, gout, and metal artifacts suppression are one example; as discussed in Schmidt and Flohr (2022), such scans are routinely used in the clinic with the image-based approach. This is not to say that the image-based decomposition with consecutive LE and HE scans is immune to patient motion, since the images must still be perfectly registered.

Although our focus has been on spatially-aligned LE and HE data, it is important to note that our methodology could be combined with principles of one-step image reconstruction (e.g. Maaß *et al* (2011), Malusek *et al* (2017), Chen *et al* (2025)) to address DE data acquisition with inconsistent rays. That is, our investigation supports robust handling of scatter and knowledge of the energy response to enable deployment of the mathematical concepts of one-step image reconstruction. However, this would not work for DE based on dual source data acquisition, because our scatter model is too simple to account for cross-scatter. In this regard, the results in Magnusson *et al* (2024) are promising.

Last, the evaluation reported here only involved data from one scanner offered by one vendor. In our early investigations (Haase *et al* 2022), we observed that the method is robust to slight changes in the scanner model. To apply our methodology to an arbitrary scanner, including scanners from other vendors, the energy response of the detector must be determined as well as the source spectrum for each detector element (to account for the bowtie filter). Once these two quantities are known, our algorithmic steps are straightforward to reproduce. Whether the same observations in image quality would be observed for all scanners is unknown; if the method did not perform as well, it could be due to important differences in the scatter signal that prevents estimating it sufficiently well with our simple model.

## 8. Conclusion

We introduced a novel method to perform material decomposition in the data domain. This method is an extension of an approach we recently introduced to improve beam hardening correction based on a single material (Haase *et al* 2022). Application of the method requires estimating and subtracting scatter with a simple model that is optimized in an object-specific manner, and it requires having access to an analytical model for the energy response of each measurement. We performed an extensive evaluation against image-based material decomposition using DE measurements obtained from consecutive axial scans. The evaluation was focused on accuracy of attenuation values, which remains a challenging problem for current DE methods. The results showed that the data-based approach often outperforms the conventional image-based approach, especially at energies below 70 keV. We also demonstrated that important improvements in performance can be seen when scanning an anthropomorphic abdomen phantom.

In future work, we plan to explore clinical utilization of this method in settings where patient motion is well-controlled. We also plan investigating its extension to commercial photon-counting CT where image-based material decomposition is currently in use, to identify if it can produce similar gains. Such an extension would have the important advantage of not being sensitive to patient motion since photon-counting CT produces the LE and HE measurements at the exact same time points.

## Data Availability

The data cannot be made publicly available upon publication because they contain commercially sensitive information. The data that support the findings of this study are available upon reasonable request from the authors.
